# Supplementary material for: N-Acetylcysteine to Reduce Kidney and Liver Injury Associated with Drug-Resistant Tuberculosis Treatment
Source: Pharmaceutics. 2025 Apr 15;17(4):516. doi: 10.3390/pharmaceutics17040516 (PMC12030172; doi:10.3390/pharmaceutics17040516)
Supplement: Supplementary file 1 [file pharmaceutics-17-00516-s001.zip › pharmaceutics-3495170-supplementary.pdf]

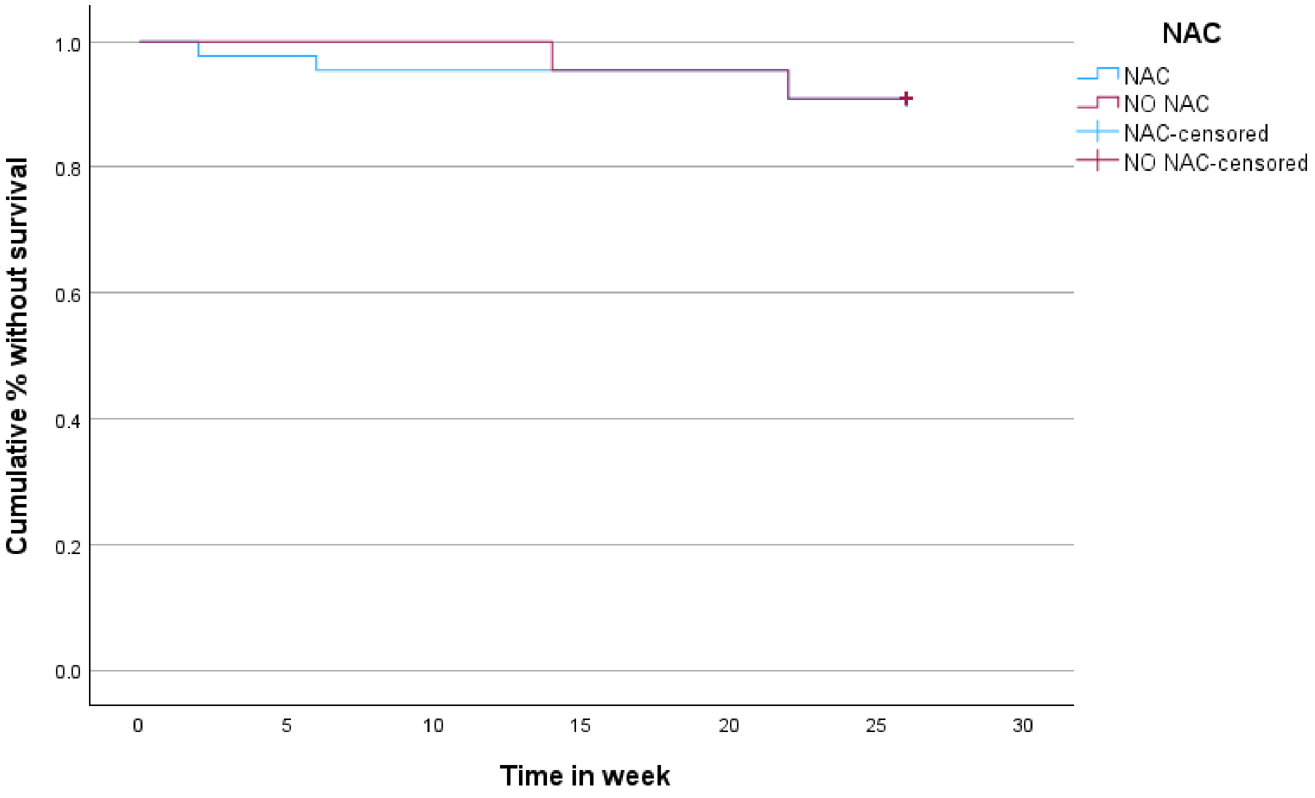

Supplemental Figure 1: liver injury time to event curve through 26 weeks with standard treatment and NAC

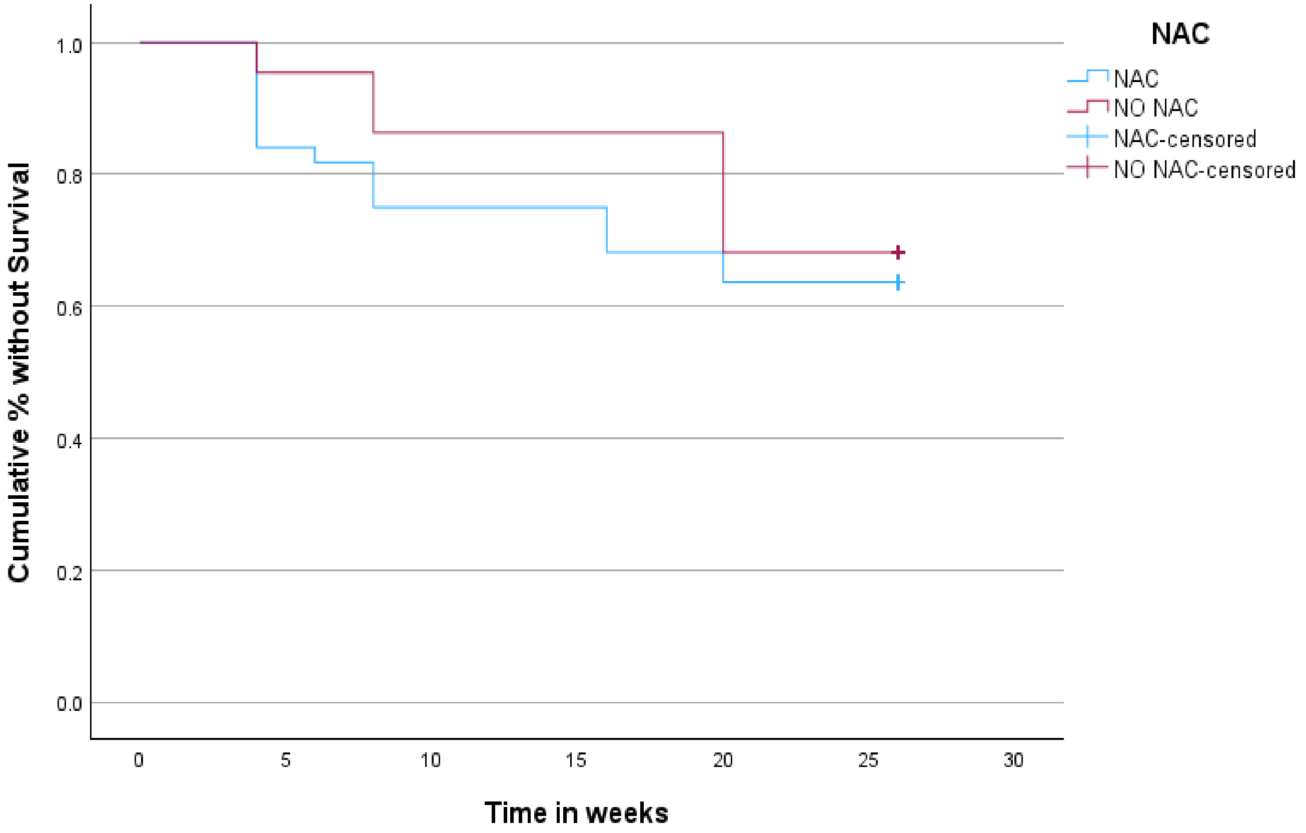

Supplemental Figure 2: anemia time to event curve through 26 weeks between standard treatment and NAC group.

Supplemental Table 1: Comparing the incidence of adverse events in the combined N-acetylcysteine vs standard treatment group (N = 66)

systems	TAE in the standard treatment group	Total patients with at least one event in the standard treatment group (N=22)	TAE in combined NAC group	Total patients with at least one event in a combined NAC group (N = 44)	TAE in all patients across	Total patients with at least one AE across (N=66)	P-value
Nervous system	**0**	0	2	2 (4.5 %)	2	2	0.549
Visual	**0**	0	2	2 (4.5%)	2	2	0.549
Endocrine	**2**	2 (9) %	1	1 (2.3%)	3	3	0.256
Gastro intestinal tract	**5**	3 (14%)	11	7 (16%)	20	10	0.281
Hepatic	**2**	2 (9%)	4	4 (9%)	6	6	1.000
Renal	**16**	10 (45%)	17	10 (22%)	33	20	0.058
Muscular skeletal	**15**	6 (27%)	43	13 (30%)	58	19	0.442
Skin	**3**	3 (14%)	1	1 (2.3%)	4	3	0.104
Hematology	**9**	7 (32%)	21	16 (36%)	30	23	0.715

Note: N-acetylcysteine (NAC), total adverse event (TAE), Analysis with Fisher exact test/χ^2
